# Retroperitoneal Tumors and Intraligamentary Pregnancy

**Published:** 1912-05

**Authors:** John M. Lee

**Affiliations:** Rochester, N. Y.


INDEXED

BUFFALO MEDICAL JOURNAL

VOL. LXVII. MAY, 1912. No, 10

### ORIGINAL COMMUNICATIONS

### Retroperitoneal Tumors and Intraligamentary Pregnancy.

By JOHN M. LEE, M.D.
Rochester, N. Y.

SO far as I know there is no very satisfactory treatment for the exceptionally large growths of these regions laid down in the text-books of our profession. For this reason surgeons are accustomed to draw upon their own experience and judgment for the management of these unfortunate cases. My own methods in this line of work may prove of value to some, therefore I will give them without further excuse or comment. There are three sites for these growths that have been of especial interest to me: First, from the cervix between the peritoneum and mucous membrane of Douglas' pouch. Second, from the uterus under the folds of the broad ligament and downward rupture of the tube between the folds of the same membrane in extrauterine pregnancy. Third, tumors of the kidneys.

I have often been surprised at the extreme displacement of the pelvic peritoneum from pregnancies and tumors which sometimes develop beneath it. Twice within two weeks in February, 1900, I operated upon enormous broad ligament cysts both of which sprang from under the right broad ligament, and hence in that respect resembled intraligamentary or extraperitoneal pregnancy. The larger tumor was estimated to weigh eighty pounds. The smaller growth also filled the abdomen. The uterus was pushed forward and to the left, the folds of both broad ligaments were stretched out and obliterated, the peritoneum was gradually lifted from the floor of the pelvis, from the rectum and from the lateral and posterior surfaces of the uterus and bladder off the sides and brim of the pelvis, the back, sides and front surfaces of the abdominal walls, and you will be surprised when I tell you that the cecum and appendix were lifted clear up under the ensiform cartilage where they were found and observed by all present. The only places where the peritoneum was ruptured were underneath the median line of the abdomen over the tumor and again just to the left of the cecum. I have enucleated many tumors from the pelvic floor and have often been surprised at the extent to which the peritoneum may be lifted, but in these cases it was so great that it scarcely seems possible, and no one who has not seen similar conditions can appreciate the extreme height to which the pelvic peritoneum may be carried by benign neoplasms.

In intra-ligamentary pregnancy at the time of spurious labor the distention is the same though less in degree, and one may see how readily the fetus may be reached by incision at the right or left of the median line and the peritoneal cavity not be opened at all; in these cases of tubal rupture downward, between the folds of the broad ligament, the ovum may develop from its original site with part of it within the tube and the other half between the folds of the mesometrion, or it may be completely extruded between these membranes which is the more frequent occurrence. Whether the fetus is entirely between the folds of the broad ligament or not it may develop until about the fifth month when secondary rupture may occur. In fact this may result shortly after the primary rupture, or there may be several ruptures in quick succession or none at all between the time the tube first gives way and term. These secondary ruptures are exceedingly hazardous, and if the mother's life is not sacrificed by the hemorrhage or the fetus does not perish, the pregnancy goes on to term within its membranes either as ligamentoperitoneal or peritoneal gestation.

Again, in rare instances the secondary rupture occurs during the eighth month and the fetus develops in the peritoneal cavity for several weeks without an enveloping membrane.

So far as I have been able to search the literature there are only four cases of this character on record: The first by Mr. T. R. Jessop referred to by Tait in his work on abdominal Surgery, Page 494; the second by Prof. James C. Wood, the third by a surgeon in the West, whose name I cannot recall, and the fourth was operated on by me in January, 1898. All of these women recovered.

My case was Mrs. W. P. S. of Rochester. There had been a primary and secondary rupture though the symptoms were not marked. The first occurred about the tenth week and the second during the eighth month. The incision showed a fully developed fetus completely extruded from the sac and transversely placed across the epigastrium with its back downward. There was not a scrap of membrane about it anywhere. When the peritoneal cavity was opened, an enormous quantity of liquor amnii rushed out with flakes of *vernix caseosa*. The incision was extended above the umbilicus when the somewhat flattened buttocks with a scrotum came in plain view. The skin resembled in color the liver and at first sight those who witnessed the operation thought these parts were really an hypertrophied liver and the child's scrotum which hung down between the folds of its buttocks so completely resembled the gall-bladder that the appearance of the above condition was complete. The tube was the site of the pregnancy for the first ten weeks, the broad ligament five or six months and the abdomen for eight months, about sixteen months in all. The placenta was spread over the broad ligament, tube, intestines and the omentum.

In all of these cases the placentae had their attachments at the site of the primary or secondary ruptures. They were widely spread over the tissues and the children all lay free in the abdomen surrounded with amniotic fluid without a bit of membrane.

If the tubal rupture is downward between the folds of the broad ligament, which on account of the anatomical relations is not so common, the pregnancy becomes intraligamentary or extraperitoneal, and as the hemorrhage is not so great the immediate chances for the patient are correspondingly better. In this unusual site the development of the ovum as in other locations may be arrested at any stage, there may be several secondary ruptures or it may go to term.

The operation, therefore, in this variety of the disease must be varied to suit the requirements: If the pregnancy is advanced sufficiently to carry the peritoneum upward the incision should be made to the right or the left of the median line near the ilium so as to reach the gestation without opening into the peritoneal cavity, then shell out the membrane, pack the sac lightly with gauze and compel it to granulate up and cicatrize over. If not able to divide the tissues at the side of the *linea alba* outside of the peritoneum, make the incision in the median line or over the most prominent part of the enlargement, then place gauze pads or towels between the tumor and abdominal wall all around, open the peritoneum of the broad ligament down to the pregnancy and extract it in such a manner, if possible, as not to soil the margins of the wound. If the cavity is aseptic, it may be sewed up with buried sutures and the abdomen closed; but if large it should be cleansed out with an antiseptic solution, if required, and the sac stitched to the peritoneum of the lower part of the abdominal wound. This forms a deep, entirely extraperitoneal cylindrical wound which may be packed lightly with gauze or drained with rubber tubes. These are both ideal methods for the treatment of infected pregnancies which result in pelvic abscesses.

When this intraligamentary pregnancy is far advanced, remove the fetus and tie the broad ligament if possible with strong catgut near the horn of the uterus, then close to where the ovarian vessels cross the iliac artery to prevent excessive hemorrhage while removing the placenta; then stitch the sac to the abdominal wound, bodily remove the membranes and quickly pack the cavity level full with moist gauze. Now pile this material up two inches thick over the opening and the borders of the abdominal wound, over which place a guttapercha tissue protective, cotton and binder.

If secondary rupture has occurred and one of those exceedingly rare cases with no sac presents, and the placenta is attached to the tube and broad ligament as is usual, ligate the ovarian vessels as above, then remove the placenta and pack the bleeding surface over with gauze as in the handkerchief dressing, and firmly hold it down till the hemorrhage is arrested; then bring the end of the gauze out through the abdominal wound and apply the above dressing.

If the intraligamentary pregnancy is low down in the vagina, this canal may be freely incised, the cavity well cleaned out and good drainage secured.

A remarkable case of neoplastic growth from beneath the upper part of the posterior abdominal peritoneum was that of Mrs. Lizzie T. She was about thirty-five years of age and the mother of several children. A large growth filled the entire abdomen, yet it was apparent that it did not spring from the internal genitalia. An accurate diagnosis as to its nature and site was quite out of the question prior to operation. A competent surgeon of the city had made an exploration by abdominal section and concluded that removal of the growth at that time was inexpedient.

The tumor, which proved to be sarcoma, grew rapidly and finally the pressure became so great that life was unbearable. and, while she knew the danger from operation was great, she desired surgical treatment even if there was but small chance for success. Accordingly on August 18th, 1903, she entered the hospital and two days later abdominal nephrectomy was performed.

The incision had to be made from the ensiform cartilage to the pubis to make sufficient room to turn out the tumor. It grew from the right kidney as was believed to be the case prior to the operation and the vessels, especially of the pedicle were of enormous size. Care was taken to avoid important tissues. The peritoneum over the dome of the tumor was incised at a convenient point, the tumor was carefully removed by enucleation and the division of the vascular adhesions between clamp on the proximal and ligature on the distal side of the growth. The pedicle with its great vessels was clamped close to the tumor and tied at two convenient places short distances from the clamp. The ureter was tied, the stump cauterized and the tumor removed.

A similar case was that of Katherine D. She entered the hospital July 15th, 1908. The tumor grew from beneath the peritoneum and completely filled the abdomen. The following day her operation was conducted in about the same manner as the foregoing case and her tumor was found on microscopic examination by a City pathologist to be also sarcoma and it grew from the right kidney.

The growths which emanated from the uterus and were classed as retroperitoneal were removed through semi-circular incisions of the peritoneum across their posterior surfaces. As the enucleation progressed it often became necessary to tie many very vascular adhesions. When the growths were finally removed all bleeding surfaces were secured and the immense wounds beneath the peritoneum closed by three or four rows of continuous catgut sutures, one upon the other. When the incised borders of the peritoneum were reached they were also brought together by a running catgut suture. During the placing of these stitches great care was taken not to wound the great aorta, the vena cava, or the intestines. Yet when necessary to bring organs in proper relations to one another or obliterate open spaces in which fluids might accumulate we did not hesitate to pass our sutures through the outer walls of intestines or even more important tissues.

In the removal of the kidneys with the exceedingly large growths the incisions through their peritoneal coverings were made in any direction that seemed suitable to avoid important organs and the wounds were closed in precisely the same way as was followed in the myomata.

The convalescences of all these cases were not unlike those of any ordinary abdominal operation except that they were required to remain in the hospital a few days longer. The first kidney case took up her work as usual after she returned home; several years later she gave birth to a fine healthy child, and a few months thereafter died. All of the other four remain well and follow their usual avocations.

